# An Exploratory Study on the Stable Radiomics Features of Metastatic Small Pulmonary Nodules in Colorectal Cancer Patients

**DOI:** 10.3389/fonc.2021.661763

**Published:** 2021-07-16

**Authors:** Caiyin Liu, Qiuhua Meng, Qingsi Zeng, Huai Chen, Yilian Shen, Biaoda Li, Renli Cen, Jiongqiang Huang, Guangqiu Li, Yuting Liao, Tingfan Wu

**Affiliations:** ^1^ Department of Radiology, First Affiliated Hospital of Guangzhou Medical University, Guangzhou, China; ^2^ Department of Radiology, Shenzhen Hospital, University of Hong Kong, Shenzhen, China; ^3^ Department of Gastrointestinal Surgery, First Affiliated Hospital of Guangzhou Medical University, Guangzhou, China; ^4^ Department of Pathology, First Affiliated Hospital of Guangzhou Medical University, Guangzhou, China; ^5^ Department of Pharmaceutical Diagnostics, GE Healthcare (China), Shanghai, China

**Keywords:** colorectal cancer, small pulmonary nodules, metastases, stable features, radiomics

## Abstract

**Objectives:**

To identify the relatively invariable radiomics features as essential characteristics during the growth process of metastatic pulmonary nodules with a diameter of 1 cm or smaller from colorectal cancer (CRC).

**Methods:**

Three hundred and twenty lung nodules were enrolled in this study (200 CRC metastatic nodules in the training cohort, 60 benign nodules in the verification cohort 1, 60 CRC metastatic nodules in the verification cohort 2). All the nodules were divided into four groups according to the maximum diameter: 0 to 0.25 cm, 0.26 to 0.50 cm, 0.51 to 0.75 cm, 0.76 to 1.0 cm. These pulmonary nodules were manually outlined in computed tomography (CT) images with ITK-SNAP software, and 1724 radiomics features were extracted. Kruskal-Wallis test was performed to compare the four different levels of nodules. Cross-validation was used to verify the results. The Spearman rank correlation coefficient is calculated to evaluate the correlation between features.

**Results:**

In training cohort, 90 features remained stable during the growth process of metastasis nodules. In verification cohort 1, 293 features remained stable during the growth process of benign nodules. In verification cohort 2, 118 features remained stable during the growth process of metastasis nodules. It is concluded that 20 features remained stable in metastatic nodules (training cohort and verification cohort 2) but not stable in benign nodules (verification cohort 1). Through the cross-validation (n=100), 11 features remained stable more than 90 times.

**Conclusions:**

This study suggests that a small number of radiomics features from CRC metastatic pulmonary nodules remain relatively stable from small to large, and they do not remain stable in benign nodules. These stable features may reflect the essential characteristics of metastatic nodules and become a valuable point for identifying metastatic pulmonary nodules from benign nodules.

## Introduction

Colorectal cancer (CRC) is the third most common malignant tumor in the world (1), and about 5% to 15% of colorectal patients are accompanied by lung metastases (2). Surgical resection of lung metastases is an optimal treatment method for CRC patients to survive long-term (3). According to previous studies, the 5-year survival time after surgery is 21% to 68% (4). Thus, a definitive diagnosis of lung metastasis is essential for clinical decision making and the improvement of prognosis. Because chest computed tomography (CT) scan is recommended for the detection of lung nodules in primary staging and postoperative surveillance (5), an increasing number of CRC patients are found to have indeterminate pulmonary nodules (IPNs), and approximately 30% of them eventually proved to be metastatic during the follow-up (6). Most of these nodules are <1 cm in diameter, single or double, and lack typical malignant signs. Other further examinations, such as positron emission tomography (PET-CT), can reflect the glucose metabolism of nodules, but small nodules (<1 cm) sometimes have no SUV elevation (7). Long-term follow-up is the most common policy to be used (8). However, the follow-up policy will bring additional costs to the patient and may delay the best treatment period. Therefore, at present, diagnosis of IPNs is a challenging dilemma for radiologists, which caused a puzzle for clinical staging, as well as the subsequent treatment.

Recently, “radiomics” has attracted the attention of doctors as a new medical imaging post-processing technology, which is a non-invasive technique and does not require additional examination. Radiomics refers to high-throughput extraction of a large number of quantitative imaging features from medical imaging images, data analysis, model building, and disease prediction, assisting doctors in making the most accurate diagnosis (9). It has been reported that texture features have a close relationship with the pathological type and pathological grade of tumors (10, 11). Nevertheless, so far, there was little published data on the diagnosis of IPNs in CRC patients on radiomics. TingDan Hu et al. was the first to conduct a study of IPNs in CRC patients based on radiomics and achieved a promising consequence by constructing prediction models (12, 13).

However, no studies have been conducted to extract the stable radiomics features of the CRC metastatic pulmonary nodules to our knowledge. In this study, we hypothesize that some radiomics features will reflect the essential characteristics of metastatic nodules and remain relatively unchanged during the growth and development of metastatic nodules, just as genes reflect the crucial characteristics of biological. Hence, this study aims to screen out the stable features of CRC metastatic pulmonary nodules with a diameter of ≤1 cm.

## Materials and Methods

### Patient

This retrospective study was approved by the ethics committee of the First Affiliated Hospital of Guangzhou Medical University, and the requirement for informed consent was waived. Our study included 260 small pulmonary nodules from 34 CRC patients (13 female/21 male; average age 60.1 ± 12.5 years; range 35–82 years) with lung metastasis between January 2012 and December 2019 from our database as training cohort and verification cohort 2. The inclusion criteria were: 1) With metastatic pulmonary nodules confirmed by histopathology or multiple metastases identified on thoracic CT. 2) The diameter of the nodule is ≤1 cm. Including the nodules found simultaneously as the primary tumor or the nodules found in the subsequent examination. Exclusion criteria: 1) The image quality is poor and cannot be used for quantitative analysis. 2) CRC patients with other malignant tumors.

In addition, our study included 60 small pulmonary nodules from 23 patients (17 males/6 females, mean age, 55.2 ± 13.7 years; range, 26–76 years) with benign pulmonary nodules between January 2012 and December 2019 in our database as verification cohort 1. The inclusion criteria were: 1) With benign pulmonary nodules confirmed by pathology or clinical follow-up for 2 years. 2) The maximum diameter of the nodule is ≤1 cm. Exclusion criteria: The image quality is poor and cannot be used for quantitative analysis.

**Figure 1:**
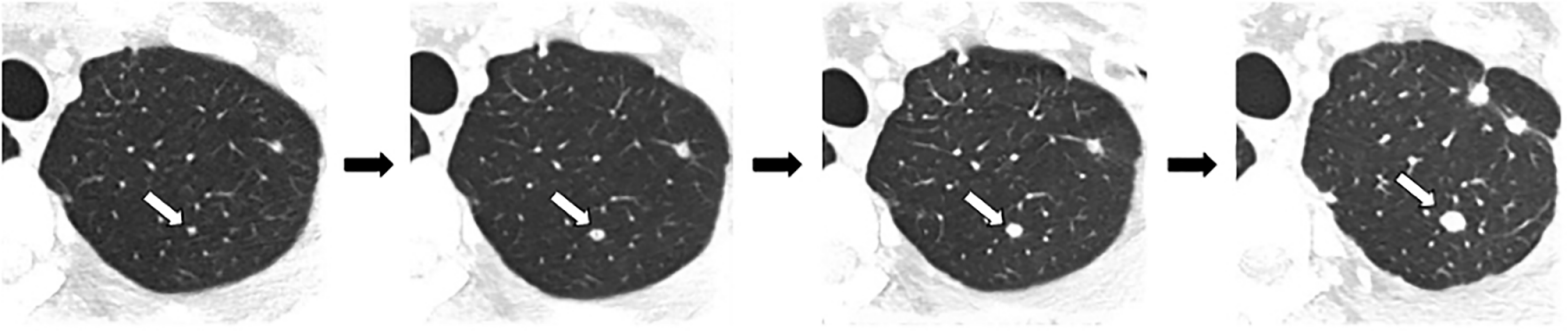
The process of nodule volume increasing on CT.

A total of 320 nodules were selected for radiomics analysis, which included training cohort (200 metastatic nodules), verification cohort 1 (60 benign nodules), and verification cohort 2 (60 metastatic nodules). In these three cohorts, we divided the nodules into four groups according to their maximum diameter respectively, including group A (0–0.25 cm), group B (0.26–0.50 cm), group C (0.51–0.75 cm), and group D (0.76–1.00 cm). **Figure 1** shows the process of nodule volume increasing on CT.

### CT Image Acquisition

All patients underwent contrast-enhanced CT with a multi-detector row CT (Siemens Somatom Definitions AS + 128 rows CT, Germany) using the following parameters: voltage, 120 kV; current, 150–200 mA; matrix, 512 × 512; scanning layer thickness, 0.60 mm; and reconstruction layer thickness, 2.0 mm. A high-pressure auto-injector was used to inject the non-ionic iodine contrast agent (Omnipaque 300, GE Healthcare, Shanghai, China) through the anterior elbow vein. The dose was 1.0 to 1.5 ml/kg, and the injection flow rate was 3 to 4 ml/s. Arterial and venous phase scans were routinely performed. The scanned image and dose report were transferred to the hospital’s picture archiving and communication system (PACS). The venous phase images were used for analysis.

### Lesion Segmentation

**Figure 2:**
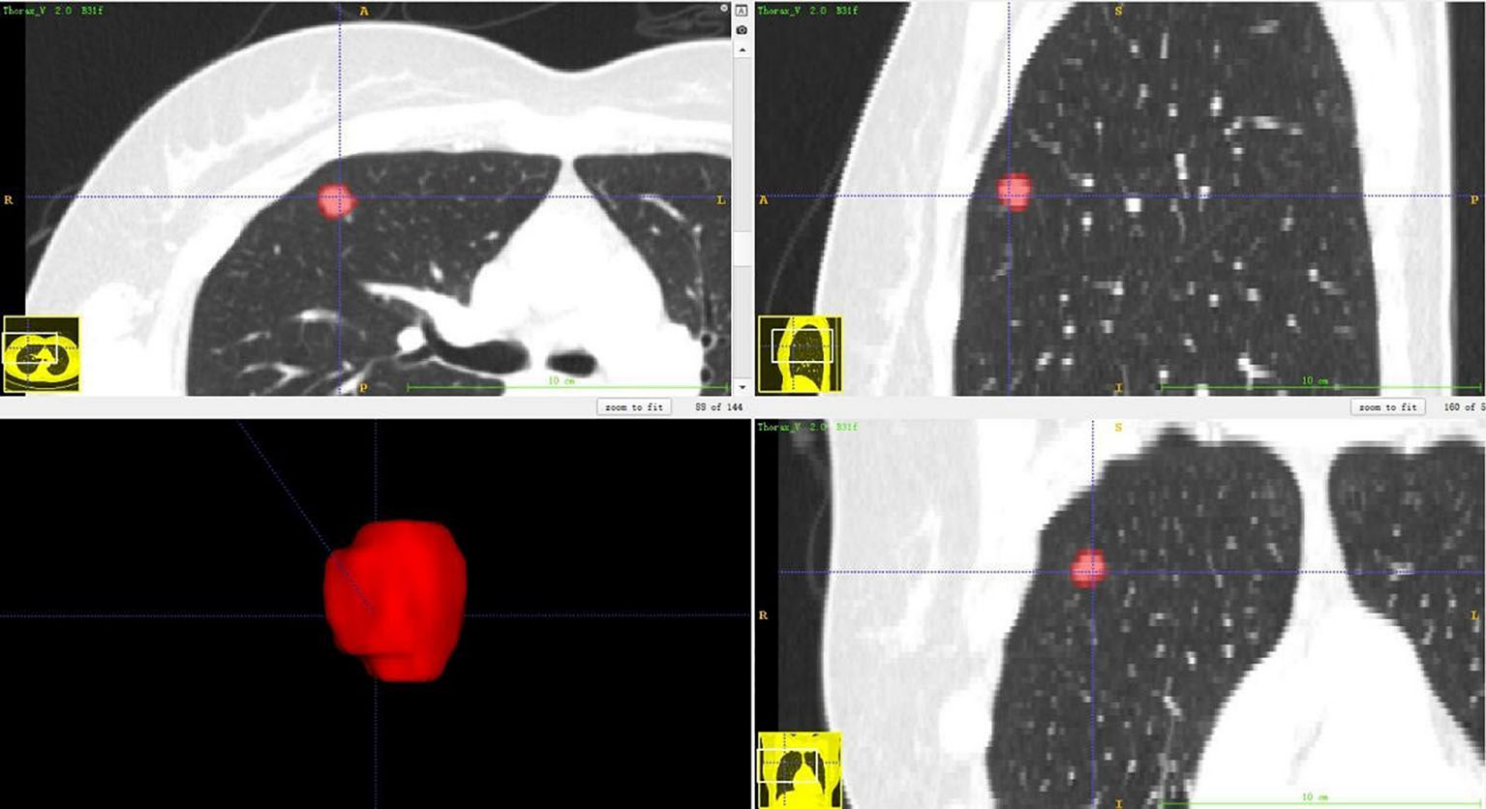
3D segmentation of pulmonary nodules by ITK-SNAP software.

The lesion segmentation was completed by two radiologists with ITK-SNAP (version 3.6.0) software. First, A radiologist of three years of lung imaging diagnosis experience delineated the lesion preliminarily, and then, a radiologist of 14 years of lung imaging diagnosis experience adjusted and confirmed the delineation of the lesion area. Both were blind to the pathological results and clinical information. Three-dimensional region of interest (ROIs) was segmented, avoiding the large blood vessels, bronchi, and pleura. **Figure 2** shows one example of 3D segmentation of pulmonary nodules by ITK-SNAP.

### Feature Extraction

All CT images and corresponding segmented ROIs were imported into the AK software (GE Healthcare) to perform image preprocessing and feature extraction. Image preprocessing methods include image standardization and image conversion. Image standardization: selected the following parameters in ImagePreprocessing: resample: X spacing, Y spacing, Z spacing were all set to 1.000 mm; intensity standardization: gray level discretization: desired minimum: 0.0, desired maximum: 255.0; grey discretization: 64. Two image conversions were performed: Co-occurrence of Local Anisotropic Gradient Orientations (CoLIAGe) and combination of Discrete wavelet transform and Local binary pattern (DWT + LBP).

Original features include 18 features from Firstorder, 23 from gray level cooccurence matrix (GlCM), 16 from gray level run length matrix (GLRLM), 16 from gray level SizeZone matrix (GlSZM), five from neighboring gray tone difference matrix (NGTDM), 13 from gray level dependence matrix (GLDM), three from shape, 13 from textural, three from normalized_radial_lengths, one from Area ratio of macroscopic contour, and one from Roughness index of boundary.

Conversion of CoLlAGe: First, compute the gradient orientation on a per-pixel basis within the ROI of lesion. Second, obtain the dominant orientations within a neighborhood of each pixel by principal component analysis. Third, calculate second-order statistics in the dominant direction.

Conversion of DWT + LBP: It is the combination of Discrete wavelet transform and Local binary pattern. DWT is to decompose the original image into four new sub-images to replace it. Each sub-image is 1/4 times the size of the original image. Four new sub-images will be generated. LBP, which encodes the local structure around each pixel. Each pixel is compared with its eight neighbors in the 3 × 3 neighborhood by subtracting the central pixel value. The strict negative value is encoded with 0, and the others are encoded with 1. The binary number is obtained and marked with its corresponding decimal value by connecting all these binary codes clockwise.

Finally, 1,724 radiomics features were extracted from every ROI of lesion. Among them, 122 features were for the original image, 1,170 for CoLIAGe, and 432 for DWT+LBP classification.

### Radiomics Analysis

**Figure 3:**
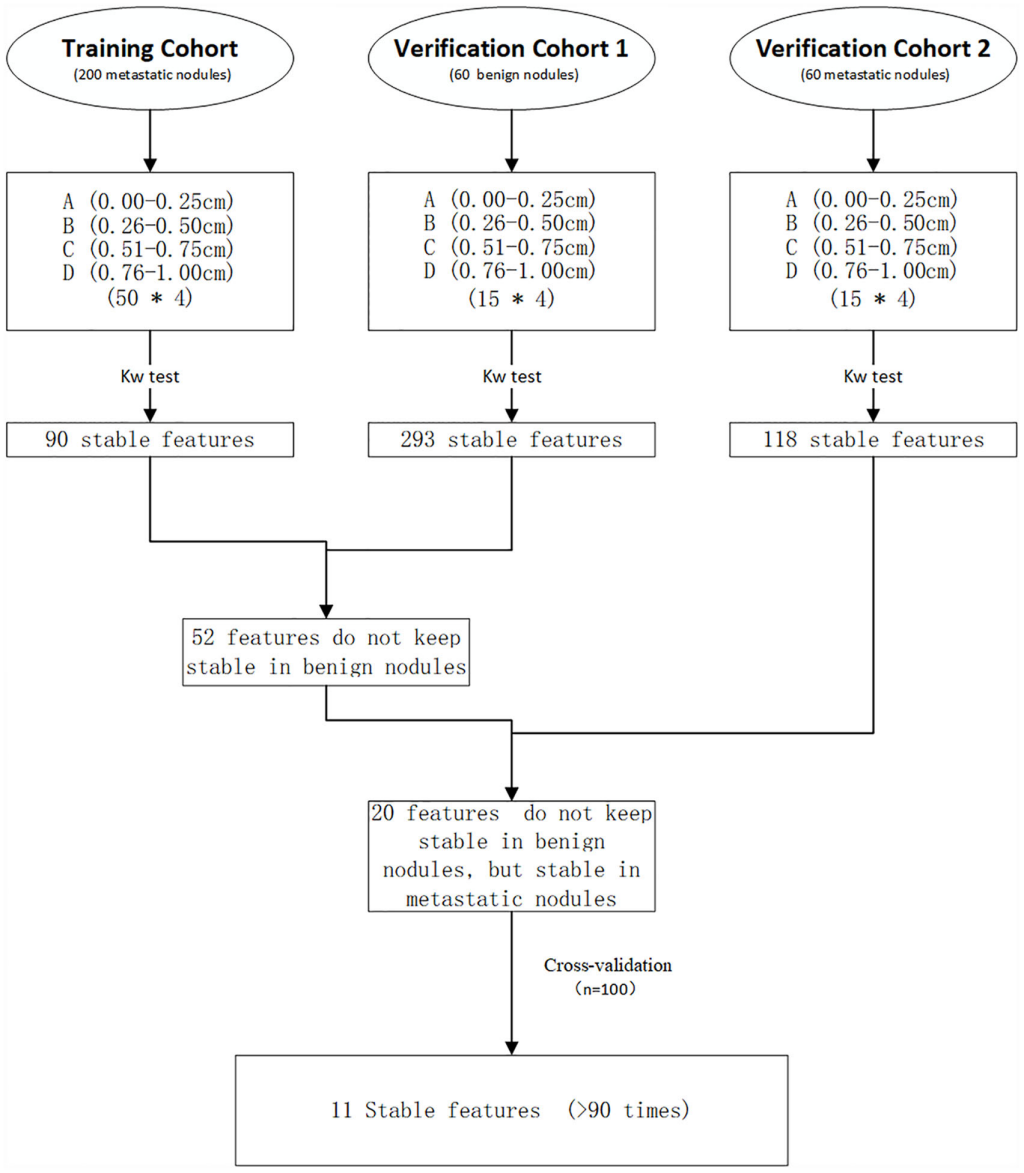
Experimental flowchart.

Radiomics features preprocessing was performed in AK software. Two hundred and sixty metastatic nodules were randomly divided into training cohort and verification cohort 2 at the ratio of 3:1. Then, we obtained the features that remained stable (no statistically significance) during the evolution of 0.25-> 0.5-> 0.75-> 1 cm in the training cohort, verification cohort 1, and verification cohort 2, respectively. Comparing the similarities and differences of these radiomics features of the three cohorts, we obtained the features that remained stable in the training cohort and did not keep stable in the verification cohort 1 (benign nodules) but remained stable in the verification cohort 2 (metastatic nodules). Besides, we performed cross-validated experiments (n = 100) that the training cohort, and verification cohort 2 randomly changed to verify the results. Features are considered as “Stable features” if they remain stable more than 90 times in cross-validation. The experimental flowchart is shown in **Figure 3**.

### Statistical Analysis

R software (version 3.6.1) was used for statistical analysis. Because all the features were non-normal distribution, radiomics features from four different levels of nodules were tested by multiple comparison tests of Kruskal-Wallis Test. A p-value <0.05 indicated statistical significance. The Spearman rank correlation coefficient is calculated to assess the correlation between the stable features and every extracted features and |ρ|>0.75, respectively.

Calculation process of Kruskal-Wallis Test: First, mix one feature data of multiple samples (Group A, B, C, D) and sort them in ascending order, assign rank 1 to the smallest observation, and rank 2 to the second smallest observation, and so on. Then, calculate the average ranks of each group of samples. finally, examine whether there are significant differences in the averages of the ranks of each group. The statistic is calculated as:

$$T=\frac{12}{N(N+1)}\sum_{i=1}^{k}\frac{R_{i}^{2}}{N_{i}}-3(N+1)$$

where *k* is the number of sample groups, *N* is the total sample size, *N_i_* is the sample size of the *i*-th group; *R_i_* is the sum of the ranks in the *i*-th group of samples.

Calculation process of Spearman rank correlation coefficient: Spearman correlation coefficient was calculated between every two features in groups A, B, C, and D, respectively. Rank the data of the two features separately and sort them in ascending order, assign rank 1 to the smallest observation, and rank 2 to the second smallest observation, and so on. The Spearman correlation coefficient is denoted by *ρ*. For the two sets of data *X* and *Y* of size *n*, convert them to grade data *x_i_*, *y_i_* (*i* = 1… …*n*). $\bar{x}$, $\bar{y}\;$present the average value of *x_i_*, *y_i_*, respectively. The correlation coefficient can be expressed as:

$$\rho =\frac{\Sigma_{i=1}^{n}(x_{i}-\bar{x})(y_{i}-\bar{y})}{\sqrt{\Sigma_{i=1}^{n}{\left(x_{i}-\bar{x}\right)}^{2}\Sigma_{i=1}^{n}}{\left(y_{i}-\bar{y}\right)}^{2}}$$

## Results

### Basic Information

The basic information of 34 CRC patients are shown in **Table 1**. The basic information of 21 patients with benign lung nodules are shown in **Table 2**.

**Table 1:** Patient characteristics for the training cohort and verification cohort 2 in the study.

Variables	Value
Patient age (range, years)	60.1 ± 12.5
Gender, n (%)	
Male	21 (61.8)
Female	13 (38.2)
Tumor location, n (%)	
Colon	18 (52.9)
Rectum	16 (47.1)
Histological grade, n (%)	
Good differentiation	1 (3.0)
Moderate differentiation	25 (73.5)
Poor differentiation	8 (23.5)
T stage of primary disease, n (%)	
1	0 (0)
2	2 (5.9)
3	18 (52.9)
4	14 (41.2)
N stage of primary disease, n (%)	
N0	12 (35.3)
N1	6 (17.6)
N2	16 (47.1)
Number of pulmonary nodules, n (%)	
Solitary	11 (32.4)
Multiple	23 (67.6)

The numbers in parentheses represent the percentage of patients in this category.

**Table 2:** Patient characteristics for the verification cohort 1 in the study.

Variables	Value
Patient age	55.2 ± 13.7
Gender, n (%)	
Male	17 (73.9)
Female	6 (26.1)
Number of pulmonary tumors, n (%)	
Solitary	13 (56.5)
Multiple	10 (43.5)

The numbers in parentheses represent the percentage of patients in this category.

### Radiomics analysis

In the initial experiment: In the colorectal cancer metastasis (training cohort), during the evolution process of 0.25-> 0.5->0.75-> 1 cm, 90 radiomics features remained unchanged relatively in total. In the benign nodules (verification cohort 1), during the evolution process of 0.25-> 0.5-> 0.75-> 1 cm, 293 radiomics features remained unchanged relatively in total. In the colorectal cancer metastasis (verification cohort 2), during the evolution process of 0.25-> 0.5-> 0.75-> 1 cm, 118 radiomics features remained unchanged relatively in total. Based on the above points, we found 20 features that remained stable in the metastasic nodules (training cohort), and they did not keep stable in the benign nodules (verification cohort 1), but remained stable in the metastatic nodules (verification cohort 2). The 20 features are list in **Table 3**. The statistical data in the initial experiment are listed in **Tables 4**–**6**, respectively.

**Table 3:** 20 stable radiomics features in the initial experiment.

Classification	Feature Parameters
“Original”	original_firstorder_Minimum
“CoLIAGe”	CoLIAGe2D_WindowSize3_Contrast_firstorder_Maximum
	CoLIAGe2D_WindowSize3_Contrast_firstorder_Range
	CoLIAGe2D_WindowSize3_Sum.Entropy_firstorder_InterquartileRange
	CoLIAGe2D_WindowSize5_Sum.Average_firstorder_90Percentile
	CoLIAGe2D_WindowSize5_Sum.Average_firstorder_Maximum
	CoLIAGe2D_WindowSize7_Entropy_firstorder_Skewness
	CoLIAGe2D_WindowSize9_Sum.Average_firstorder_Mean
	CoLIAGe2D_WindowSize9_Sum.Average_firstorder_Median
	CoLIAGe2D_WindowSize9_Sum.Average_firstorder_RootMeanSquared
	CoLIAGe2D_WindowSize9_Sum.Average_firstorder_Skewness
	CoLIAGe2D_WindowSize11_Sum.Average_firstorder_10Percentile
	CoLIAGe2D_WindowSize11_Sum.Average_firstorder_Mean
	CoLIAGe2D_WindowSize11_Sum.Average_firstorder_Median
	CoLIAGe2D_WindowSize11_Sum.Average_firstorder_RootMeanSquared
	CoLIAGe2D_WindowSize11_Sum.Average_firstorder_Skewness
“DWT + LBP”	wavelet.HHL_lbp.3D.m1_firstorder_Mean
	wavelet.HHL_lbp.3D.m1_firstorder_Skewness
	wavelet.LLL_lbp.3D.m1_firstorder_Kurtosis
	wavelet.LLL_lbp.3D.k_firstorder_Skewness

Take CoLIAGe2D_WindowSize3_Contrast_firstorder_Maximum as an example, CoLIAGe2D represents the type of image conversion, WindowSize3 represents the parameters for conversion, first order represents the feature type, and Maximum represents the name of the feature.

**Table 4:** Statistical data of training cohort.

Feature parameters	t-A	t-B	t-C	t-D	t-*P*
original_firstorder_Minimum	-869.000 (-914.750, -873.200)	-865.000 (-888.750, -862.600)	-864.500 (-890.000, -864.860)	-877.500 (-893.750, -862.280)	0.501
CoLIAGe2D_WindowSize3_Contrast_firstorder_Maximum	208.000 (189.000,216.440)	205.000 (192.000,210.000)	200.500 (192.000,207.280)	206.500 (192.000,217.040)	0.621
CoLIAGe2D_WindowSize3_Contrast_firstorder_Range	206.500 (188.000,212.620)	205.000 (192.000,209.860)	200.500 (192.000,207.280)	206.500 (192.000,217.040)	0.678
CoLIAGe2D_WindowSize3_Sum.Entropy_firstorder_InterquartileRange	1.000 (1.000,0.910)	1.000 (1.000,1.000)	1.000 (1.000,0.960)	1.000 (1.000,1.020)	0.569
CoLIAGe2D_WindowSize5_Sum.Average_firstorder_90Percentile	40.000 (23.600,33.998)	41.650 (38.250,40.538)	38.900 (38.000,39.588)	40.750 (38.000,40.300)	0.649
CoLIAGe2D_WindowSize5_Sum.Average_firstorder_Maximum	45.000 (30.000,37.760)	46.000 (46.000,45.700)	46.000 (46.000,45.760)	46.000 (46.000,46.180)	0.736
CoLIAGe2D_WindowSize7_Entropy_firstorder_Skewness	0.000 (0.000,0.327)	0.001 (-0.216,0.106)	0.101 (-0.096,0.069)	0.167 (-0.004,0.154)	0.107
CoLIAGe2D_WindowSize9_Sum.Average_firstorder_Mean	9.312 (2.000,23.854)	30.827 (20.322,29.834)	24.310 (19.780,24.585)	24.174 (22.793,24.243)	0.117
CoLIAGe2D_WindowSize9_Sum.Average_firstorder_Median	9.000 (2.000,23.690)	34.750 (14.250,30.420)	24.000 (17.000,24.390)	25.000 (22.000,24.080)	0.106
CoLIAGe2D_WindowSize9_Sum.Average_firstorder_RootMeanSquared	12.547 (2.000,24.157)	33.793 (25.300,32.671)	27.992 (23.949,28.149)	27.799 (26.458,27.703)	0.078
CoLIAGe2D_WindowSize9_Sum.Average_firstorder_Skewness	0.000 (0.000,0.036)	-0.186 (-0.987,-0.273)	0.022 (-0.309,0.031)	0.014 (-0.147,0.056)	0.131
CoLIAGe2D_WindowSize11_Sum.Average_firstorder_10Percentile	2.000 (2.000,23.200)	14.000 (2.000,19.988)	2.500 (2.000,9.166)	4.000 (2.000,4.964)	0.059
CoLIAGe2D_WindowSize11_Sum.Average_firstorder_Mean	3.000 (2.000,23.689)	33.907 (16.544,30.457)	22.110 (14.589,24.220)	23.789 (21.965,24.486)	0.160
CoLIAGe2D_WindowSize11_Sum.Average_firstorder_Median	3.000 (2.000,23.750)	37.250 (7.250,29.980)	19.000 (5.250,23.380)	24.000 (19.000,23.740)	0.210
CoLIAGe2D_WindowSize11_Sum.Average_firstorder_RootMeanSquared	3.000 (2.000,23.781)	36.267 (23.436,33.075)	26.642 (20.469,28.204)	28.271 (26.581,28.719)	0.128
CoLIAGe2D_WindowSize11_Sum.Average_firstorder_Skewness	0.000 (0.000,-0.004)	0.000 (-1.137,-0.365)	0.185 (-0.775,-0.057)	0.085 (-0.204,0.055)	0.714
wavelet.HHL_lbp.3D.m1_firstorder_Mean	11.008 (10.379,10.911)	10.917 (10.711,10.926)	10.848 (10.757,10.855)	10.858 (10.800,10.857)	0.327
wavelet.HHL_lbp.3D.m1_firstorder_Skewness	0.009 (-0.133,0.000)	0.008 (-0.032,0.015)	0.014 (-0.005,0.015)	0.011 (-0.015,0.011)	0.936
wavelet.LLL_lbp.3D.m1_firstorder_Kurtosis	1.951 (1.714,2.075)	2.128 (1.746,2.246)	1.695 (1.463,1.985)	1.929 (1.702,2.195)	0.095
wavelet.LLL_lbp.3D.k_firstorder_Skewness	0.680 (0.0250,1.289)	1.039 (0.568,1.025)	0.749 (0.606,0.814)	0.775 (0.592,0.922)	0.236

Group t-A, t-B, t-C, t-D represent four different levels of nodules from training cohort: 0-0.25 cm, 0.26-0.50 cm, 0.51-0.75 cm, and 0.76-1.00 cm respectively. Statistical data are expressed as the median (quartile).

**Table 5:** Statistical data of verification cohort 1.

Feature parameters	v1-A	v1-B	v1-C	v1-D	v1-*P*
original_firstorder_Minimum	-831.000 (-843.500, -825.000)	-848.000 (-877.500, -845.067)	-862.000 (-881.500, -860.467)	-882.000 (-917.000, -866.600)	0.047
CoLIAGe2D_WindowSize3_Contrast_firstorder_Maximum	186.000 (132.000,176.800)	185.000 (177.000,184.200)	193.000 (183.500,200.933)	228.000 (211.500,227.867)	0.009
CoLIAGe2D_WindowSize3_Contrast_firstorder_Range	185.000 (132.000,172.867)	185.000 (177.000,184.133)	193.000 (183.500,200.933)	228.000 (211.500,227.867)	0.006
CoLIAGe2D_WindowSize3_Sum.Entropy_firstorder_InterquartileRange	1.000 (1.000,0.883)	1.000 (1.000,1.133)	1.000 (0.000,0.600)	1.000 (1.000,1.200)	0.003
CoLIAGe2D_WindowSize5_Sum.Average_firstorder_90Percentile	36.000 (31.500,34.167)	39.000 (34.600,37.593)	40.800 (38.050,39.813)	42.400 (38.050,41.867)	0.007
CoLIAGe2D_WindowSize5_Sum.Average_firstorder_Maximum	44.000 (38.000,40.267)	45.000 (44.000,42.800)	46.000 (46.000,45.200)	47.000 (46.000,46.600)	<0.001
CoLIAGe2D_WindowSize7_Entropy_firstorder_Skewness	0.253 (-0.012,0.273)	-0.296 (-0.432, -0.144)	-0.091 (-0.228, -0.027)	0.111 (-0.030,0.092)	0.008
CoLIAGe2D_WindowSize9_Sum.Average_firstorder_Mean	10.800 (4.757,17.740)	32.015 (21.499,31.717)	30.708 (23.428,29.444)	25.727 (21.244,25.366)	0.0235
CoLIAGe2D_WindowSize9_Sum.Average_firstorder_Median	8.000 (2.000,16.433)	37.000 (22.500,33.733)	35.000 (19.000,30.533)	26.000 (20.000,25.333)	0.022
CoLIAGe2D_WindowSize9_Sum.Average_firstorder_RootMeanSquared	14.224 (5.559,19.568)	34.278 (25.681,33.695)	34.245 (27.502,32.718)	30.566 (24.622,29.006)	0.028
CoLIAGe2D_WindowSize9_Sum.Average_firstorder_Skewness	0.528 (0.000,0.595)	-0.490 (-1.542,-0.724)	-0.571 (-1.196,-0.478)	-0.170 (-0.306,-0.038)	0.013
CoLIAGe2D_WindowSize11_Sum.Average_firstorder_10Percentile	2.000 (2.000,12.800)	20.000 (7.250,23.760)	14.000 (4.500,18.413)	4.000 (3.000,7.033)	0.049
CoLIAGe2D_WindowSize11_Sum.Average_firstorder_Mean	6.061 (2.048,15.688)	38.195 (23.630,33.521)	34.415 (19.887,31.551)	26.633 (21.030,25.422)	0.015
CoLIAGe2D_WindowSize11_Sum.Average_firstorder_Median	3.000 (2.000,15.167)	44.000 (22.250,34.233)	44.000 (13.500,32.533)	29.000 (15.000,25.267)	0.033
CoLIAGe2D_WindowSize11_Sum.Average_firstorder_RootMeanSquared	6.863 (2.059,16.603)	39.831 (27.274,35.155)	38.093 (25.095,34.065)	30.774 (25.324,29.391)	0.018
CoLIAGe2D_WindowSize11_Sum.Average_firstorder_Skewness	0.000 (0.000,0.990)	-0.857 (-1.554,-0.623)	-0.702 (-1.647,-0.449)	-0.260 (-0.554,-0.014)	0.032
wavelet.HHL_lbp.3D.m1_firstorder_Mean	10.983 (10.817,10.958)	10.944 (10.666,11.004)	10.626 (10.43,10.565)	10.915 (10.784,10.904)	0.011
wavelet.HHL_lbp.3D.m1_firstorder_Skewness	0.002 (-0.101,0.008)	0.002 (-0.102,-0.012)	0.078 (0.026,0.096)	-0.001 (-0.027,-0.002)	0.038
wavelet.LLL_lbp.3D.m1_firstorder_Kurtosis	2.276 (2.085,2.685)	3.005 (2.290,3.969)	1.82 (1.424,1.788)	2.058 (1.852,2.597)	<0.001
wavelet.LLL_lbp.3D.k_firstorder_Skewness	1.563 (1.295,1.948)	1.222 (0.979,1.468)	0.829 (0.548,0.814)	0.839 (0.614,0.958)	<0.001

Group v1-A, v1-B, v1-C, v1-D represent four different levels of nodules from verification cohort 1: 0-0.25 cm, 0.26-0.50 cm, 0.51-0.75 cm, and 0.76-1.00 cm respectively. Statistical data are expressed as the median (quartile).

**Table 6:** Statistical data of verification cohort 2.

Feature parameters	v2-A	v2-B	v2-C	v2-D	v2-*P*
original_firstorder_Minimum	-851.000 (-866.000,-845.800)	-879.000 (-902.500,-879.600)	-880.000 (-895.000,-871.200)	-859.000 (-891.000,-862.533)	0.056
CoLIAGe2D_WindowSize3_Contrast_firstorder_Maximum	208.000 (186.500,212.667)	197.000 (188.500,201.733)	205.000 (192.000,203.533)	205.000 (192.000,210.067)	0.670
CoLIAGe2D_WindowSize3_Contrast_firstorder_Range	208.000 (185.500,211.600)	197.000 (188.000,201.467)	205.000 (192.000,203.533)	205.000 (192.000,210.067)	0.676
CoLIAGe2D_WindowSize3_Sum.Entropy_firstorder_InterquartileRange	1.000 (1.000,0.917)	1.000 (1.000,0.867)	1.000 (1.000,1.050)	1.000 (1.000,1.133)	0.136
CoLIAGe2D_WindowSize5_Sum.Average_firstorder_90Percentile	35.200 (32.150,36.967)	38.500 (36.900,39.527)	41.800 (39.450,40.987)	43.000 (40.100,41.987)	0.211
CoLIAGe2D_WindowSize5_Sum.Average_firstorder_Maximum	45.000 (36.500,40.733)	45.000 (44.500,45.333)	46.000 (45.500,46.000)	46.000 (46.000,46.467)	0.144
CoLIAGe2D_WindowSize7_Entropy_firstorder_Skewness	0.359 (0.105,0.663)	0.346 (-0.317,0.133)	0.025 (-0.210,0.032)	0.028 (0.009,0.102)	0.0755
CoLIAGe2D_WindowSize9_Sum.Average_firstorder_Mean	42.148 (2.000,28.343)	23.075 (12.246,25.304)	27.837 (21.483,26.064)	25.387 (23.969,25.063)	0.856
CoLIAGe2D_WindowSize9_Sum.Average_firstorder_Median	48.000 (2.000,29.600)	20.000 (7.000,24.067)	29.000 (19.000,26.967)	25.500 (24.250,26.000)	0.709
CoLIAGe2D_WindowSize9_Sum.Average_firstorder_RootMeanSquared	43.054 (2.000,28.656)	28.538 (17.357,28.452)	31.405 (25.858,29.257)	29.177 (27.813,28.891)	0.841
CoLIAGe2D_WindowSize9_Sum.Average_firstorder_Skewness	0.000 (0.000 to −0.058)	0.206 (−0.095 to 0.277)	−0.209 (−0.499 to −0.034)	−0.060 (−0.211 to −0.059)	0.466
CoLIAGe2D_WindowSize11_Sum.Average_firstorder_10Percentile	48.000 (2.000,28.733)	2.000 (2.000,18.800)	6.000 (2.000,12.593)	3.400 (2.000,3.827)	0.294
CoLIAGe2D_WindowSize11_Sum.Average_firstorder_Mean	48.000 (2.000,29.400)	12.396 (4.205,22.826)	30.670 (18.045,26.898)	26.044 (22.887,25.162)	0.746
CoLIAGe2D_WindowSize11_Sum.Average_firstorder_Median	48.000 (2.000,29.600)	2.000 (2.000,21.200)	35.000 (11.500,27.700)	27.000 (20.250,26.567)	0.646
CoLIAGe2D_WindowSize11_Sum.Average_firstorder_RootMeanSquared	48.000 (2.000,29.431)	18.529 (6.632,24.915)	34.368 (24.237,29.828)	30.358 (28.175,29.711)	0.750
CoLIAGe2D_WindowSize11_Sum.Average_firstorder_Skewness	0.000 (0.000 to −0.132)	0.000 (0.000,0.561)	−0.574 (−1.316 to −0.586)	−0.191 (−0.339 to −0.052)	0.318
wavelet.HHL_lbp.3D.m1_firstorder_Mean	10.727 (9.980,10.650)	11.110 (10.705,10.988)	10.943 (10.791,10.897)	10.739 (10.649,10.764)	0.214
wavelet.HHL_lbp.3D.m1_firstorder_Skewness	0.061 (−0.004,0.087)	−0.011 (−0.076 to 0.004)	0.023 (−0.048 to 0.011)	0.037 (−0.002 to 0.025)	0.221
wavelet.LLL_lbp.3D.m1_firstorder_Kurtosis	2.011 (1.778,1.978)	2.116 (1.623,2.11)	1.755 (1.456,1.894)	1.914 (1.456,1.873)	0.648
wavelet.LLL_lbp.3D.k_firstorder_Skewness	1.264 (0.106,1.638)	0.800 (0.500,1.059)	0.852 (0.557,0.851)	0.848 (0.478,1.024)	0.992

Group v2-A, v2-B, v2-C, v2-D represent four different levels of nodules from verification cohort 2: 0–0.25 cm, 0.26–0.50 cm, 0.51–0.7 5 cm, and 0.76 1.00 cm, respectively. Statistical data are expressed as the median (quartile).

Through 100 times of cross-validation, 11 features remain stable more than 90 times, and 19 features remain stable more than 80 times. The features are list in **Table 7**. The definition of these features is attached to the supplementary material.

**Table 7:** The stable radiomic features in Cross-validation (n=100).

classification	19 features keep stable in Cross-validation more than 80 times	11 features keep stable in Cross-validation more than 90 times
“Original”	original_firstorder_Minimum	original_firstorder_Minimum
“CoLIAGe”	CoLIAGe2D_WindowSize3_Contrast_firstorder_Maximum	CoLIAGe2D_WindowSize3_Contrast_firstorder_Maximum
	CoLIAGe2D_WindowSize3_Contrast_firstorder_Range	CoLIAGe2D_WindowSize3_Contrast_firstorder_Range
	CoLIAGe2D_WindowSize3_Sum.Entropy_firstorder_InterquartileRange	CoLIAGe2D_WindowSize3_Sum.Entropy_firstorder_InterquartileRange
	CoLIAGe2D_WindowSize5_Sum.Average_firstorder_90Percentile	CoLIAGe2D_WindowSize5_Sum.Average_firstorder_90Percentile
	CoLIAGe2D_WindowSize5_Sum.Average_firstorder_Maximum	_
	CoLIAGe2D_WindowSize5_Entropy_firstorder_InterquartileRange	_
	CoLIAGe2D_WindowSize7_Sum.Average_firstorder_Kurtosis	_
	CoLIAGe2D_WindowSize9_Sum.Average_firstorder_Mean	_
	CoLIAGe2D_WindowSize9_Sum.Average_firstorder_Median	_
	CoLIAGe2D_WindowSize9_Sum.Average_firstorder_RootMeanSquared	_
	CoLIAGe2D_WindowSize9_Sum.Average_firstorder_Skewness	CoLIAGe2D_WindowSize9_Sum.Average_firstorder_Skewness
	CoLIAGe2D_WindowSize11_Sum.Average_firstorder_Mean	_
	CoLIAGe2D_WindowSize11_Sum.Average_firstorder_Median	CoLIAGe2D_WindowSize11_Sum.Average_firstorder_Median
	CoLIAGe2D_WindowSize11_Sum.Average_firstorder_RootMeanSquared	CoLIAGe2D_WindowSize11_Sum.Average_firstorder_RootMeanSquared
	CoLIAGe2D_WindowSize11_Sum.Average_firstorder_Skewness	CoLIAGe2D_WindowSize11_Sum.Average_firstorder_Skewness
“DWT + LBP”	wavelet.HHL_lbp.3D.m1_firstorder_Mean	_
	wavelet.HHL_lbp.3D.m1_firstorder_Skewness	wavelet.HHL_lbp.3D.m1_firstorder_Skewness
	wavelet.LLL_lbp.3D.k_firstorder_Skewness	wavelet.LLL_lbp.3D.k_firstorder_Skewness

Take CoLIAGe2D_WindowSize3_Contrast_firstorder_Maximum as an example, CoLIAGe2D represents the type of image conversion, WindowSize3 represents the parameters for conversion, first order represents the feature type, and Maximum represents the name of the feature.

Twelve pairs of features were chosen using Spearman correlation and |ρ|>0.75, including 7 Stable features and 10 other extracted features. There is a high correlation(|ρ|>0.90) between a pair of Stable features. Eleven extracted features are relatively highly correlated (|ρ|>0.75) with Stable features, and 4 of them have poor stability. These features and statistics are list in **Table 8**.

**Table 8:** Twelve pairs of features with correlation coefficient (|*ρ*|) > 0.75.

Stable features screened out	Related features	Group	*ρ*
original_firstorder_Minimum(99)	original_firstorder_10Percentile(0)	A	0.965
		B	0.923
		C	0.898
		D	0.752
CoLIAGe2D_WindowSize3_Contrast_firstorder_Range(96)	CoLIAGe2D_WindowSize3_Contrast_firstorder_Maximum(97)	A	0.963
		B	0.998
		C	1.000
		D	1.000
CoLIAGe2D_WindowSize5_Sum.Average_firstorder_90Percentile(91)	CoLIAGe2D_WindowSize5_Sum.Average_firstorder_RootMeanSquared(0)	A	0.970
		B	0.865
		C	0.788
		D	0.817
CoLIAGe2D_WindowSize11_Sum.Average_firstorder_Median(96)	CoLIAGe2D_WindowSize9_Sum.Average_firstorder_Mean(88)	A	0.948
		B	0.944
		C	0.954
		D	0.809
CoLIAGe2D_WindowSize11_Sum.Average_firstorder_Median(96)	CoLIAGe2D_WindowSize9_Sum.Average_firstorder_RootMeanSquare(84)	A	0.948
		B	0.943
		C	0.949
		D	0.763
CoLIAGe2D_WindowSize11_Sum.Average_firstorder_Median(96)	CoLIAGe2D_WindowSize11_Sum.Average_firstorder_Mean(90)	A	0.991
		B	0.964
		C	0.989
		D	0.963
CoLIAGe2D_WindowSize11_Sum.Average_firstorder_Median(96)	CoLIAGe2D_WindowSize11_Sum.Average_firstorder_RootMeanSquared(91)	A	0.991
		B	0.962
		C	0.980
		D	0.941
CoLIAGe2D_WindowSize11_Sum.Average_firstorder_RootMeanSquared(91)	CoLIAGe2D_WindowSize9_Sum.Average_firstorder_Mean(88)	A	0.956
		B	0.989
		C	0.972
		D	0.825
CoLIAGe2D_WindowSize11_Sum.Average_firstorder_RootMeanSquared(91)	CoLIAGe2D_WindowSize9_Sum.Average_firstorder_RootMeanSquare(84)	A	0.956
		B	0.982
		C	0.971
		D	0.808
CoLIAGe2D_WindowSize11_Sum.Average_firstorder_RootMeanSquared(91)	CoLIAGe2D_WindowSize11_Sum.Average_firstorder_90Percentile(54)	A	0.991
		B	0.881
		C	0.950
		D	0.775
CoLIAGe2D_WindowSize11_Sum.Average_firstorder_RootMeanSquared(91)	CoLIAGe2D_WindowSize11_Sum.Average_firstorder_Mean(90)	A	1.000
		B	1.000
		C	0.994
		D	0.973
wavelet.LLL_lbp.3D.k_firstorder_Skewness(95)	wavelet.LLL_lbp.3D.k_firstorder_Kurtosis(0)	A	0.912
		B	0.915
		C	0.771
		D	0.851

The first column is the Stable features that remained stable more than 90 times in Cross-validation (n=100). The second column is features that are related to Stable features. The number in () represents the number of times that the features remained stable in cross-validation (n=100).

## Discussion

Our result shows that during the evolution of CRC metastases (200 cases) from small to large, a total of 90 features remained unchanged relatively, of which 20 features did not keep stable in the benign nodules of the verification cohort 1, but kept stable in the metastases of the verification cohort 2. Eleven features remain stable for more than 90 times in cross-validation (n=100), and these 11 features are all included in the scope of the 20 features in the initial experiment. This result validates our initial hypothesis that there may be inherent radiomic features in the metastatic pulmonary nodules from colorectal cancer. These stable radiomics features may be the potential tools we are looking for to diagnosis lung metastases of CRC and assist clinical treatment decision making.

Whether IPNs are metastases determines the M stage of colorectal cancer, as well as the treatment options. CT scan intuitively shows the morphological characteristics of the pulmonary lesion. However, at present, conventional CT can only accurately diagnose multiple metastases, Large nodules (14), or ground-glass nodules (ground-glass nodules rarely occur in metastatic cancer). Most small solid nodules (≤1 cm) are difficult to identify whether metastatic or benign nodules because they both tended to be round or oval with a smooth contour (15, 16). Contrast-enhanced CT and PET/CT with FDG are hard to assess nodules smaller than this size either. Biopsy is considered to be the “gold standard” for the diagnosis of lung metastases. Still, the application of biopsy is clinically limited because it is an invasive procedure that may cause complications after sampling. Besides, it cannot evaluate the histopathological features of nodules as a whole. Although Most IPNs are benign, lung metastases still need to be identified as early as possible because they may develop rapidly. Diagnosis of early lung metastases has a positive impact on clinical practice, and timely detection of them will benefit patients in the long term. Only a small percentage of scholars specifically conducted a radiomics study concerning the indeterminate nodules smaller than 1 cm (13, 17). Therefore, how to manage uncertain small pulmonary nodules of CRC patients becomes an important issue at present. Considering these factors, we chose to analyze the features of small nodules(≤1 cm) in this study.

As is known to all, benign and malignant tumor are completely different in genetic characteristics. It determines different cell morphology and biological behavior, reflecting different Histopathological structures and imaging representation (18). In order to obtain more useful information from image data of lesion, high-throughput extraction radiology images can provide valuable assistance (19). A retrospective study reported that radiomics features can discriminate the primary lung cancer from granulomatous nodules reached an area under the curve (AUC) of 0.90 (17). However, up to now, using radiomics features to predict IPNs for CRC has only been investigated in a few studies. TingDan Hu et al. recently developed the nomograms produced by combining radiomics features and clinical risk factors that have good discrimination ability and accuracy for metastasis prediction (12, 13). It indicates that using radiomics tools to diagnose lung metastasis of colorectal cancer is feasible. This study was based on a new idea that some radiomics features of nodules with different pathologies may remain unchanged like genes. We tried to find the relatively stable features from metastatic nodules as” “radiomics markers”, which may give guidance to clinical diagnosis. The conventional process of most radiomics studies is to find the features that can best distinguish metastasis from non-metastasis, and incorporate it into the model, such as logistic regression model, artificial neural network, and random forest, then finally, verify the model (20). By contrast, this study employed the method of hypothesis testing (Kruskal-Wallis test) to conduct this exploration study. Kruskal-Wallis test (21) is a nonparametric statistical test that can be used to evaluate whether two or more samples come from the same distribution. Thus, we utilized this statistical test method to seek the radiomics commonality of metastatic nodules, that is, to seek the inherent features of radiomics of them. Our study finally screened out 11 stable features from three categories, including “Original”, “CoLIAGe” and “DWT + LBP” by AK software. Origin features (22) are the earliest and important medical imaging diagnostic features, including traditional first-order statistics, shape descriptors, and texture descriptors as GLCM and GLRLM, etc. Studies (23) have confirmed that they have a certain ability to distinguish benign and malignant nodules. CoLlAGe (24) is a new radiomics descriptor proposed by Prateek Prasanna et al. It captures high-order co-occurrence patterns of local gradient tensors at the voxel level from medical imaging and can distinguish subtle pathological differences and disease phenotypes from similar morphological manifestations. They found that adenocarcinoma lesions have a greater density of CoLlAGe entropy compared with granuloma on computed tomography. Januar AdiPutra1 proposed to develop some new feature extraction and selection algorithms to improve the accuracy of classification. He combined wavelet and LBP (25), which able to divide breast tissue into normal or abnormal, and the performance of this scheme can produce high accuracy of 92.71%. Most of the stable features obtained in our research are “CoLlAGe”. A total of 11 features keep stable in Cross-validation (100 times) more than 90 times. Eight of them belong to the “CoLIAGe”, one of them belong to “original” and two of them belongs to “DWT + LBP”. Metastatic nodules and benign nodules have different phenotypes in histopathology. However, CT appearance performance of small metastatic nodules such as shape, edge are very similar to small benign nodules. So the original features extracted such as “shape” are difficult to distinguish metastatic nodules from benign nodules. The texture feature can distinguish subtle pathological differences to a certain extent, but it only analyzes the difference in the overall intensity pattern of nodules. On the contrary, “CoLIAGe” is to identify the difference of local entropy pattern, which reflects the subtle local difference in microstructure. So, it can distinguish subtle pathology differences from similar overall texture and appearance in imaging.

We found that 12 pairs of features with a correlation coefficient >0.75 in Group A, B, C, and D. One pair of them both are Stable features. Seven features related to Stable features lacked stability slightly. Four features (original_firstorder_10Percentile, CoLIAGe2D_WindowSize5_Sum.Average_firstorder_RootMeanSquared, CoLIAGe2D_WindowSize11_Sum.Average_firstorder_90Percentile, wavelet.LLL_lbp.3D.k_firstorder_Kurtosis) related to Stable features were found to lack stability obviously, which is not surprising because their correlation with Stable features is relatively low.

Our study has some limitations. First, this is a single-center experiment, using the same CT machine and images of the same period to avoid the difference in image data. Still, it is not conducive for the promotion of the results. Second, there is a bias in the sample because this is a retrospective study, which may limit the accuracy of the results. Third, we used the criterion of 2-year stability for diagnosing benign lesions on thoracic CT do not have pathological confirmation. Fourth, manually delineating ROI may not be as repeatable as semi-automatic delineation and automatic delineation. Last, this study is still in the initiatory exploration stage, so the results still need a lot of data to confirm and further data mining.

Nowadays, radiomics is developing towards a promising direction as a non-invasive post-processing technology. However, the current radiomics approaches may be lacking in repeatability and reproducibility. Standardized image acquisition and reconstruction, multi-center data support, data sharing, and expansion of sample size will be necessary in the future.

## Conclusions

In summary, this study shows that certain radiomics features of metastatic nodules are stable during the evolution process and do not keep stable in benign nodules, which provide another potential approach to the study of lung metastasis and the development of artificial intelligent diagnosis.

## Data Availability Statement

The original contributions presented in the study are included in the article/supplementary material. Further inquiries can be directed to the corresponding author.

## Ethics Statement

The studies involving human participants were reviewed and approved by the ethics committee of the First Affiliated Hospital of Guangzhou Medical University. The patients/participants provided their written informed consent to participate in this study.

## Author Contributions

QM, QZ, HC, and TW designed the study. CL, YS, BL, RC, JH, and GL collected the data. CL and QM Segmented the lesion. TW performed the data extraction and analyzed the data. TW and YL carried out the statistical analysis. CL wrote the manuscript. QM and YL reviewed and modified the manuscript. HC made the final revision to the article. All authors contributed to the article and approved the submitted version.

## Funding

The work of Huai Chen was supported by the Natural Science Foundation of Guangdong Province, China (2019A1515011382).

## Conflict of Interest

The authors declare that the research was conducted in the absence of any commercial or financial relationships that could be construed as a potential conflict of interest.
